# Analysis of the global burden of diabetes and attributable risk factor in children and adolescents across 204 countries and regions from 1990 to 2021

**DOI:** 10.3389/fendo.2025.1587055

**Published:** 2025-09-08

**Authors:** Yu Hu, Tingting He, Yu Zhang, Yang Long, Chenlin Gao, Yong Xu

**Affiliations:** ^1^ Department of Endocrinology and Metabolism, The Affiliated Hospital of Southwest Medical University, Luzhou, Sichuan, China; ^2^ Southwest Medical University, Luzhou, Sichuan, China; ^3^ Cardiovascular and Metabolic Diseases Key Laboratory of Luzhou, Affiliated Hospital of Southwest Medical University, Luzhou, Sichuan, China; ^4^ Sichuan Clinical Research Center for Nephropathy, Affiliated Hospital of Southwest Medical University, Luzhou, Sichuan, China; ^5^ Metabolic Vascular Disease Key Laboratory of Sichuan Province, Luzhou, Sichuan, China

**Keywords:** diabetes, children and adolescents, global burden of disease, risk factors, subgroup analysis

## Abstract

**Background:**

While middle-aged and elderly individuals account for the majority of diabetes cases, recent years have witnessed a rising incidence of diabetes, particularly type 2 diabetes, among younger populations. This study aims to examine global trends in diabetes among children and adolescents (<20 years) across 204 countries and regions.

**Methods:**

This study draws primarily from the 2021 GBD database, offering an in-depth analysis of trends in the incidence, mortality, and disability-adjusted life years (DALYs) of diabetes in children and adolescents from 1990 to 2021, with further subgroup analyses conducted by sex, age, and specific regions. Furthermore, we investigated the potential risk factors contributing to diabetes-related mortality in children and adolescents.

**Results:**

Between 1990 and 2021, the global incidence of diabetes among children and adolescents exhibited a steady upward trend. By 2021, the incidence of diabetes had risen by approximately 94.0% compared to 1990, while both mortality and DALYs experienced a notable decline. Subgroup analyses region revealed that death rates were generally lower in regions with a high sociodemographic index (SDI) compared to those with a low SDI and low income. Additionally, high fasting blood glucose, along with extreme temperature conditions (both high and low), were identified as the three major risk factors contributing to diabetes-related mortality in children and adolescents.

**Conclusions:**

While diabetes-related mortality in children and adolescents has declined over time, the sharp increase in incidence indicates that it is emerging as a major threat to global child and adolescent health.

## Highlights

• Why did we undertake this study?
Although diabetes predominantly affects middle-aged and older adults, there has been a notable increase in the incidence of diabetes, especially type 2 diabetes, among younger populations in recent years.
• What is the sp*ecific question(s) we wanted to answer?*
Global Epidemiological Trends of Diabetes Among Children and Adolescents from 1990 to 2021.
• What did we find?
The burden of diabetes among children and adolescents varies markedly by sex and geographic region. While the diabetes-related mortality rate among children and adolescents has declined over time, the incidence has risen sharply.
Elevated fasting blood glucose and high and low temperatures are the three principal risk factors for diabetes-related mortality.
• What are the implications of our findings?
Diabetes is emerging as a significant global health threat to children and adolescents. Consequently, healthcare institutions must devise more cost-effective and targeted strategies to alleviate the adverse effects of diabetes on children and adolescents.

## 1 Introduction

Diabetes is a severe chronic condition primarily characterized by elevated blood glucose levels, typically resulting from absolute or relative insulin deficiency. Estimates from the 2019 Global Burden of Disease (GBD) study indicate that diabetes has emerged as the eighth leading cause of death and disability, affecting approximately 460 million people worldwide in 2019 (1). Furthermore, the International Diabetes Federation (IDF) reported in the 2021 Diabetes Atlas that the global prevalence of diabetes reached 537 million individuals in 2021. Projections indicate that by 2030, the number of individuals with diabetes may increase to 643 million and escalate to an alarming 783 million by 2045, with global healthcare expenditures potentially surpassing $105.4 billion (2). This highlights the immense burden that diabetes imposes on healthcare systems (3–5). The Non-Communicable Diseases Risk Factor Collaboration (NCD-RisC) estimated in 2016 that the probability of achieving the global objective of halting the rise in diabetes prevalence by 2025 was less than 1% for females and even lower for males (6).

Although diabetes can be categorized into various subtypes based on its underlying pathological mechanisms, it is predominantly classified as type 1 and type 2 diabetes (7). Type 1 diabetes is commonly recognized as an autoimmune disorder mediated by T cells, usually emerging in childhood and reaching its peak incidence around 14 years of age (8, 9). Krolewski et al. (10) observed a secondary peak in incidence around age 50, though this accounts for only 7% of cases. Conversely, type 2 diabetes is influenced by a complex interplay of genetic, dietary, and lifestyle factors and typically manifests after the age of 40 (11). Recent studies have highlighted a rising incidence of diabetes among younger individuals, indicating that the condition is increasingly posing a significant threat to an expanding cohort of young people (12). Despite advancements in prevention strategies, including enhanced basic healthcare infrastructure and increased availability of insulin and antidiabetic medications, these measures have yet to effectively curb the rising incidence and the trend of earlier onset of diabetes (13). Consequently, this study utilizes the most recent data from GBD 2021 to examine the evolving epidemiological trends of diabetes among children and adolescents (<20 years) across 204 countries from 1990 to 2021. Additionally, it aims to analyze risk factors associated with diabetes-related mortality, thereby aiding relevant organizations in developing more effective prevention and treatment policies to counteract the trend of earlier onset diabetes.

## 2 Method

### 2.1 Data source

The GBD 2021 study examined over 370 diseases and injuries across 204 countries and regions, offering comprehensive data on incidence, mortality, and Disability-Adjusted Life Years (DALYs) (14, 15). All data are derived from reputable public databases and have undergone rigorous screening processes to ensure data integrity (16).

Deaths and mortality rates are primarily estimated using the cause-of-death ensemble model, whereas prevalence and incidence are assessed using the Bayesian meta-regression model. Disability weights are conceptualized as indicators of the severity of health loss or non-fatal disability, whereas DALYs represent the aggregate of years lost due to health impairment from onset to death (17).

The Socio-Demographic Index (SDI) is a composite metric that reflects a country’s developmental status, incorporating lag-distributed income per capita, the average educational attainment of individuals aged 15 and older, and the total fertility rate among those under 25. It has a significant association with health outcomes. SDI values span from 0 to 1, with 0 indicating the lowest level of development and 1 denoting the highest level (18). Based on SDI values, the 204 countries and regions are stratified into five categories: high SDI, high-middle SDI, middle SDI, low-middle SDI, and low SDI regions. Furthermore, patients are classified into five age subgroups: under 1 year, 2 – 4 years, 5 – 9 years, 10 – 14 years, and 15 – 19 years.

### 2.2 Estimation of risk factor

We identified 66 specific attributable risk factors for evaluation, including particulate pollution, high temperature, low temperature, lead exposure, smoking, secondhand smoke exposure, high consumption of red meat, high sodium intake, low fiber intake, low fruit consumption, low vegetable intake, and elevated fasting blood glucose, as defined by prior research (19). Ultimately, relevant data were available only for high temperature, low temperature, and elevated fasting blood glucose, leading the subsequent attribution analysis to concentrate on these three risk factors.

### 2.3 Definition of diabetes

In the 2021 Global Burden of Disease study, diabetes is classified based on the International Classification of Diseases, Tenth Revision (ICD - 10), specifically codes E10 and E11.

### 2.4 Statistical analyses

To examine the relationship between trends in incidence and mortality rates and levels of social development, we computed the Pearson correlation coefficient concerning SDI values. Statistical analyses, including t-tests and analysis of variance (ANOVA), were performed using GraphPad Prism (version 10.0.0 for Windows) to compare incidence rates, mortality rates, and DALYs across different genders, age groups, and regions. The Autoregressive Integrated Moving Average (ARIMA) model was constructed to predict the changing trends in the incidence and mortality rates of diabetes among children and adolescents worldwide over the next 15 years. Unlike conventional linear regression, the ARIMA model offers a more accurate computational method that captures the dynamics of time series while incorporating additional relevant information (20). All hypothesis tests were conducted with a two-sided approach, and a significance level was set at P < 0.05.

## 3 Result

### 3.1 Global trends and incidence of diabetes in children and adolescents

**Figure 1:**
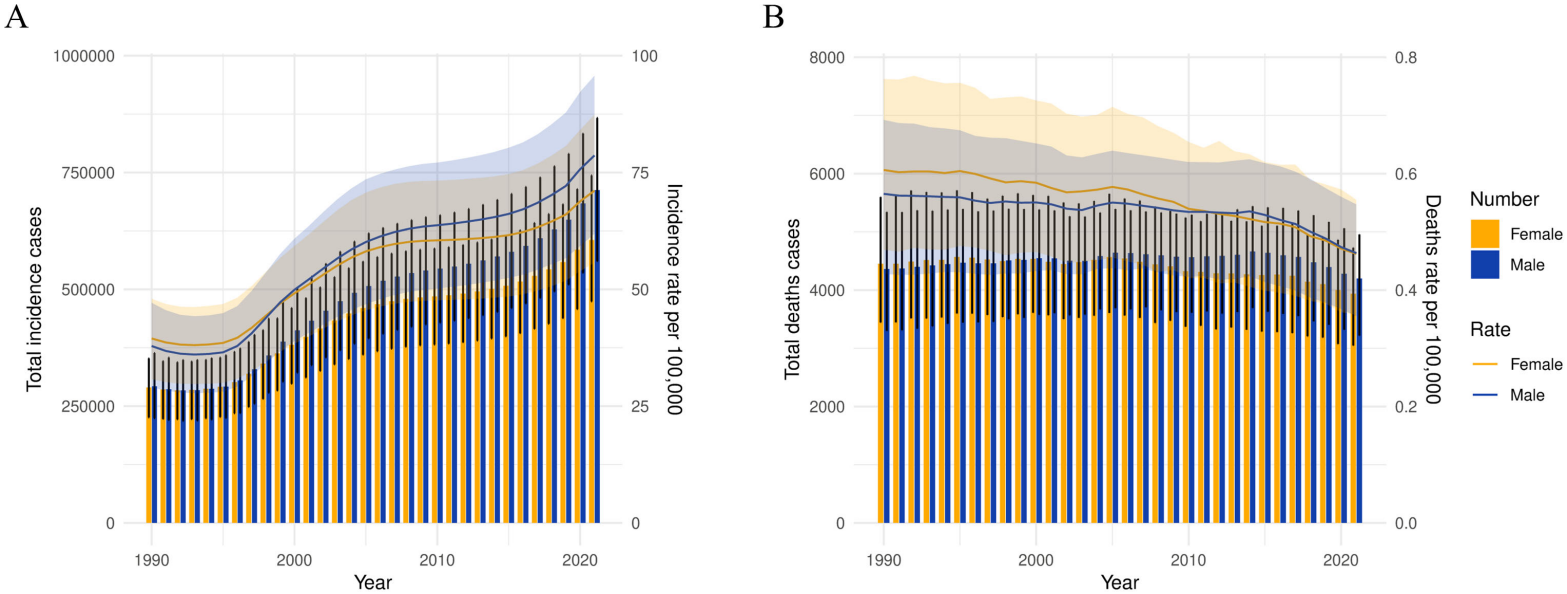
The change trends of diabetes’s incidence cases, and deaths from 1990 to 2021. **(A)** The change trends of incidences, **(B)** the change trends of deaths. Blue bars represent males and orange bars represent females.

**Figure 2:**
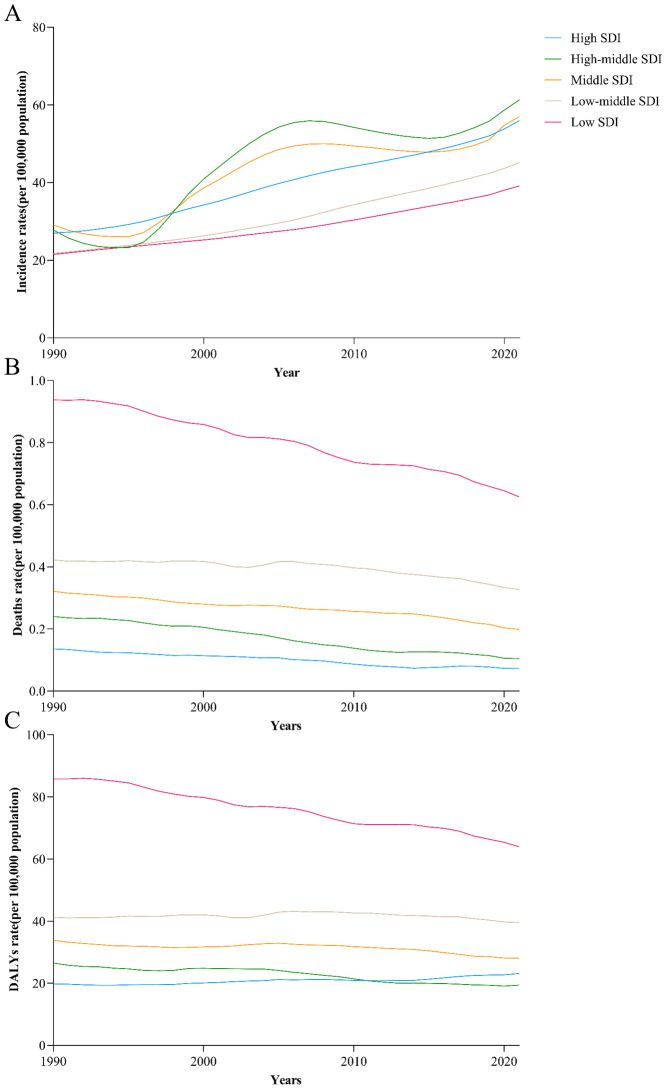
Trends from 1990 to 2021 in the death rate of diabetes in five SDI regions. **(A)** The change trends of incidences, **(B)** the change trends of deaths, **(C)** the change trends of DALYs.

**Figure 3:**
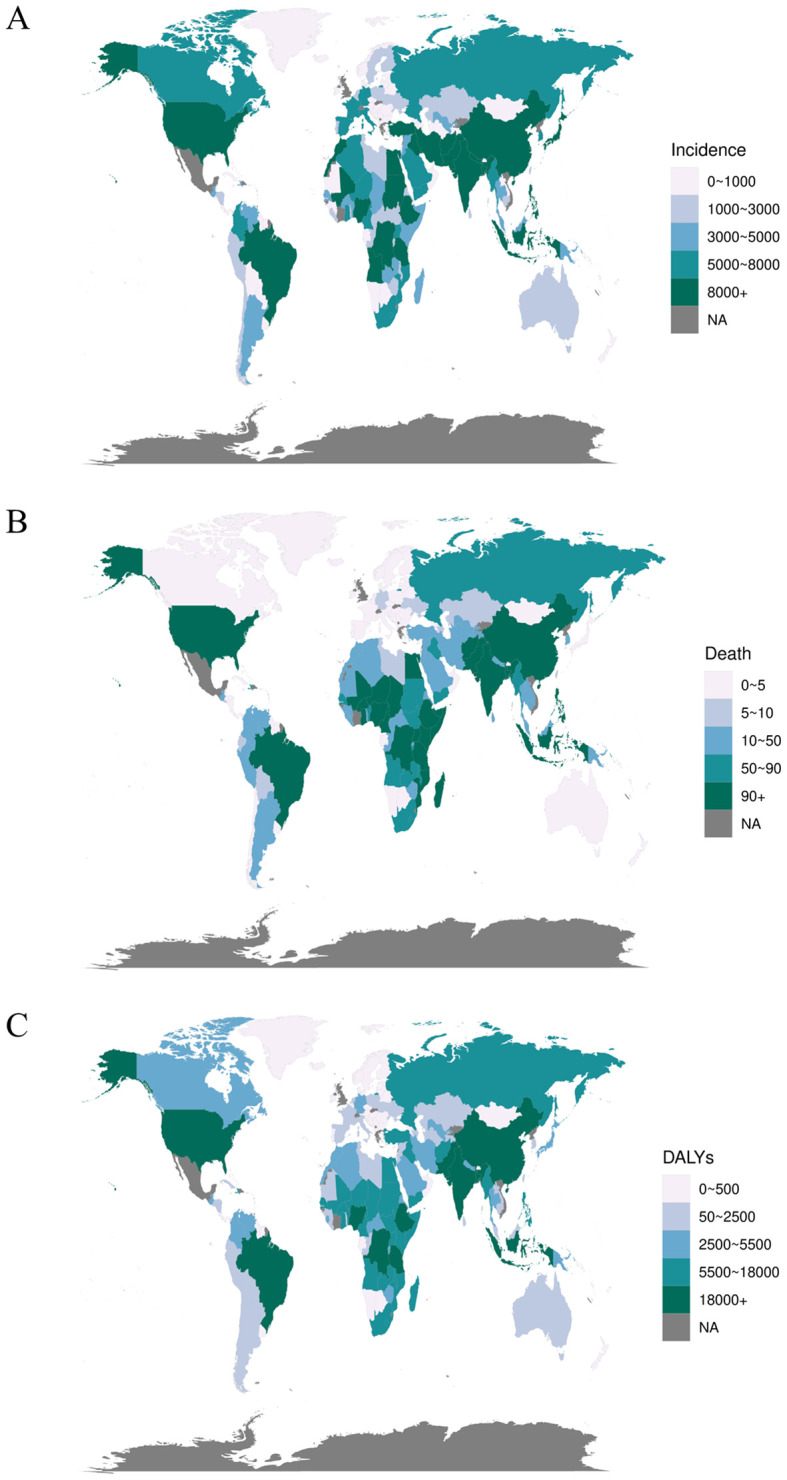
The global disease burden of diabetes in 204 countries or territories. **(A)** The incidence cases of 204 countries or territories in 2021, **(B)** the deaths of 204 countries or territories in 2021, and **(C)** the DALYs of 204 countries or territories in 2021.

**Figure 4:**
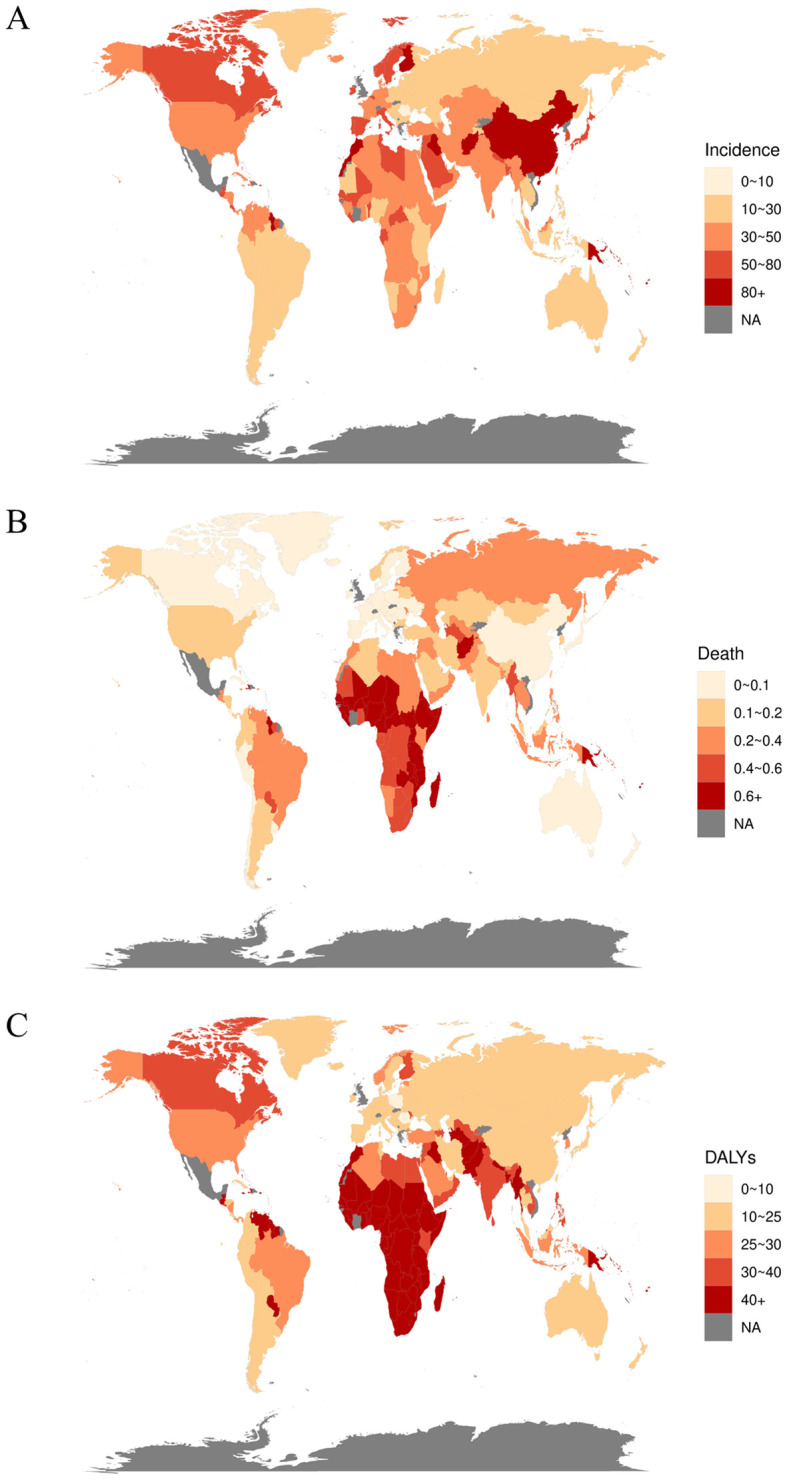
The rates of diabetes in 204 countries or territories. **(A)** The incidence rate of 204 countries or territories in 2021, **(B)** the death rate of 204 countries or territories in 2021, and **(C)** the DALY rate of 204 countries or territories in 2021.

Globally, the incidence of diabetes among children and adolescents has demonstrated a significant annual increase over the past 32 years, rising from 25.77 per 100,000 in 1990 to 49.99 per 100,000 in 2021, reflecting an approximate 94.0% increase. The total number of diabetes cases escalated from 581,949 in 1990 to 1,317,669 in 2021. The prevalence of both type 1 and type 2 diabetes has increased, with type 2 diabetes rates surpassing those of type 1 diabetes (**Supplementary Figure S1**). Notably, although the absolute number of cases is higher in males, the incidence rate for females exceeded that of males before 2020 (**Table 1**, **Figure 1A**). At the SDI level, diabetes incidence trends upward across all five SDI regions. The Low SDI region exhibits the lowest burden, while the High-middle SDI region shows the highest burden, with an incidence rate of 61.3 (95% UI: 47.71 - 75.84) (**Table 1**, **Figure 2A**, **Supplementary Figures S3**, **S4**). Geographically, Oceania reports the highest incidence rates, increasing from 48.29 per 100,000 in 1990 to 101.00 per 100,000 in 2021. The North Africa and Middle East, as well as East Asia regions, experienced the most substantial increases in incidence rates (**Table 2**). At the national level, China and India report the highest numbers of diabetes cases, with figures rising from 177.6×10^3^ and 93.2×10^3^ in 1990 to 342.1×10^3^ and 243.0×10^3^ in 2021, respectively. Meanwhile, Pakistan’s number of cases increased from seventh place in 1990 (14.87×10^3^) to third place in 2021 (53.7×10^3^) (**Figures 3A**, **4A**, **Supplementary Tables S1**, **S2**).

**Table 1:** The incidence rate of diabetes in 1990/2021.

Group	1990 Rate per 10^3^ (95% UI)	2021 Rate per 10^3^ (95% UI)
Overall	25.77 (19.82, 31.61)	49.99 (39.30, 60.89)
Sex		
Male	25.24 (19.35,31.40)	52.43 (41.26,63.79)
Female	26.32 (20.51,31.97)	47.40 (37.13,58.22)
Disease subtype		
Type 1 diabetes	9.00 (6.97,11.48)	10.43 (7.93,13.31)
Type 2 diabetes	16.77 (10.88,22.85)	39.56 (29.21,50.75)
SDI		
High SDI	26.96 (21.80,31.91)	55.97 (44.49,66.90)
High-middle SDI	27.76 (20.56,36.04)	61.31 (47.71,75.84)
Middle SDI	29.09 (21.66,36.85)	56.99 (44.64,69.71)
Low-middle SDI	21.73 (16.82,26.60)	45.12 (34.69,56.50)
Low SDI	21.49 (25.40,17.54)	39.15 (31.10,47.76)
Region		
Central Asia	17.48 (13.66,21.28)	28.95 (22.49,35.11)
Central Europe	10.33 (8.20,12.75)	14.01 (10.73,17.57)
Eastern Europe	12.82 (9.75,15.99)	17.53 (13.20,22.43)
Latin America and Caribbean	25.55 (19.46,31.73)	39.59 (29.68,49.95)
North Africa and Middle East	19.71 (15.54,23.92)	53.43 (41.46,65.88)
South Asia	23.71 (18.15,29.55)	51.16 (38.79,65.24)
East Asia	39.61 (27.86,52.94)	101.27 (78.71,125.95)
Oceania	48.29 (37.46,58.91)	101.00 (79.33,124.87)
Southeast Asia	18.69 (14.31,23.17)	26.21 (19.80,32.85)
Sub-Saharan Africa	20.56 (16.97,24.24)	33.65 (26.90,40.98)
World Bank Income Level		
World Bank High Income	26.23 (21.12,31.08)	52.20 (41.26,62.30)
World Bank Upper Middle Income	31.94 (23.38,41.89)	70.65 (55.46,87.55)
World Bank Lower Middle Income	21.30 (16.52,26.31)	41.92 (32.30,52.63)
World Bank Low Income	22.34 (18.61,26.06)	40.03 (32.12,48.24)

UI, uncertainty intervals.

**Table 2:** The DALY of diabetes in 1990/2021.

Group	1990 Rate per 10^3^ (95% UI)	2021 Rate per 10^3^ (95% UI)
Overall	39.45 (32.91,47.02)	37.93 (30.76,46.13)
Sex		
Male	38.28 (29.64,46.74)	38.20 (30.16,46.69)
Female	40.69 (32.17,53.36)	37.65 (29.51,47.26)
Disease subtype		
Type 1 diabetes	30.96 (25.35,37.95)	23.60 (18.47,28.16)
Type 2 diabetes	8.50 (6.66,11.16)	14.33 (10.96,19.30)
SDI		
High SDI	19.79 (16.38,24.35)	23.16 (16.85,32.16)
High-middle SDI	26.47 (22.14,32.37)	19.41 (15.07,25.47)
Middle SDI	33.87 (29.35,41.13)	28.07 (22.85,35.10)
Low-middle SDI	41.22 (34.00,49.89)	39.52 (32.01,48.18)
Low SDI	85.79 (61.66,108.32)	63.96 (49.67,78.27)
Region		
Central Asia	27.82 (24.61,31.67)	30.76 (26.02,36.43)
Central Europe	12.79 (10.97,15.20)	9.80 (7.27,13.23)
Eastern Europe	20.38 (18.62,22.93)	20.65 (18.14,24.09)
Latin America and Caribbean	46.91 (42.69,52.12)	35.34 (29.85,42.54)
North Africa and Middle East	51.73 (40.10,77.55)	38.14 (30.75,50.03)
South Asia	34.20 (27.84,42.22)	34.44 (26.80,43.91)
East Asia	26.98 (21.69,34.75)	17.99 (12.67,25.12)
Oceania	70.25 (50.73,88.17)	80.57 (61.47,102.53)
Southeast Asia	33.99 (27.47,39.74)	28.77 (24.28,34.19)
Sub-Saharan Africa	89.91 (63.39,114.79)	66.09 (49.59,82.04)
World Bank Income Level		
World Bank High Income	20.04 (16.70,24.55)	22.60 (16.57,31.34)
World Bank Upper Middle Income	31.95 (27.33,38.48)	23.90 (19.02,30.63)
World Bank Lower Middle Income	41.38 (33.51,50.43)	40.52 (32.47,48.90)
World Bank Low Income	92.58 (66.58,118.51)	65.12 (51.01,79.83)

UI, uncertainty intervals.

### 3.2 Global trends and death of diabetes in children and adolescents

Among the global population of children and adolescents, the diabetes-related death rate has exhibited a general annual decline over the past 32 years, decreasing from 0.39 per 100,000 in 1990 to 0.31 per 100,000 in 2021. Correspondingly, the number of deaths has decreased from 8,813 to 8,134. Deaths associated with type 1 diabetes remain substantially higher than those associated with type 2 diabetes (**Supplementary Figure S2**); however, the death rate for type 2 diabetes has risen from 0.06 per 100,000 in 1990 to 0.08 per 100,000 in 2021. The diabetes death rate is comparable between males and females, both standing at 0.31 per 100,000 (**Table 3**, **Figure 1B**). At the SDI level, diabetes-related death rates across the five SDI regions demonstrate a generally decreasing trend. The death rate is lowest in the High SDI regions (0.07, 95% UI: 0.07 – 0.08) and highest in the Low SDI regions (0.63, 95% UI: 0.46 – 0.76) (**Table 3**, **Figure 2B**, **Supplementary Figures S3**, **S4**). Subgroup analysis by geographical region reveals that Oceania exhibits the highest death rate and an increasing trend, rising from 0.73 per 100,000 in 1990 to 0.77 per 100,000 in 2021. In contrast, East Asia has experienced the most significant reduction in death rate, decreasing by 74% relative to 1990 (**Table 3**). At the national level, India reports the highest number of diabetes-related deaths, with 1.32×10³ cases in 1990 and 0.96×10³ cases in 2021. Conversely, China’s diabetes-related deaths have decreased from the second highest in 1990 (1.02×10³ cases) to the ninth position in 2021 (0.19×10³ cases) (**Figures 3B**, **4B**, **Supplementary Tables S3**, **S4**).

**Table 3:** The death of diabetes in 1990/2021.

Group	1990 Rate per 10^3^ (95% UI)	2021 Rate per 10^3^ (95% UI)
Overall	0.39 (0.33,0.46)	0.31 (0.25,0.36)
Sex		
Male	0.38 (0.29,0.46)	0.31 (0.24,0.36)
Female	0.40 (0.31,0.51)	0.31 (0.24,0.37)
Disease subtype		
Type 1 diabetes	0.33 (0.26,0.40)	0.23 (0.17,0.27)
Type 2 diabetes	0.06 (0.05,0.07)	0.08 (0.07,0.09)
SDI		
High SDI	0.14 (0.13,0.14)	0.07 (0.07,0.08)
High-middle SDI	0.24 (0.21,0.29)	0.10 (0.10,0.12)
Middle SDI	0.32 (0.28,0.38)	0.20 (0.18,0.22)
Low-middle SDI	0.42 (0.35,0.51)	0.33 (0.27,0.38)
Low SDI	0.94 (0.66,1.20)	0.63 (0.46,0.76)
Region		
Central Asia	0.28 (0.25,0.30)	0.28 (0.25,0.32)
Central Europe	0.11 (0.10,0.11)	0.05 (0.05,0.05)
Eastern Europe	0.21 (0.20,0.21)	0.19 (0.18,0.20)
Latin America and Caribbean	0.47 (0.44,0.50)	0.28 (0.25,0.32)
North Africa and Middle East	0.54 (0.42,0.85)	0.31 (0.25,0.42)
South Asia	0.33 (0.27.0.40)	0.24 (0.20,0.28)
East Asia	0.23 (0.19,0.30)	0.06 (0.05,0.08)
Oceania	0.73 (0.50,0.96)	0.77 (0.58,1.01)
Southeast Asia	0.35 (0.29,0.42)	0.27 (0.23,0.31)
Sub-Saharan Africa	0.98 (0.68,1.27)	0.66 (0.46,0.81)
World Bank Income Level		
World Bank High Income	0.14 (0.14,0.15)	0.07 (0.07.0.08)
World Bank Upper Middle Income	0.30 (0.26,0.35)	0.15 (0.13,0.16)
World Bank Lower Middle Income	0.43 (0.34,0.51)	0.35 (0.28,0.40)
World Bank Low Income	1.01 (0.70,1.31)	0.64 (0.48,0.77)

UI, uncertainty intervals.

### 3.3 Global trends and DALY of diabetes in children and adolescents

Among the global population of children and adolescents, the DALY rate has generally declined over the past 32 years, decreasing from 39.45 per 100,000 in 1990 to 37.83 per 100,000 in 2021. However, the total DALYs have risen from 891.1×10³ years to 999.9×10³ years. Compared to 1990, the DALY rate for type 1 diabetes decreased by 2021, whereas the DALY rate for type 2 diabetes increased. Nonetheless, the DALY rate for type 1 diabetes remains higher overall, at 23.60 per 100,000 compared to 14.33 per 100,000 for type 2 diabetes (**Supplementary Figure S3**). The DALY rate for males has remained relatively stable, whereas the DALY rate for females appears to be a significant factor in the overall decrease in the DALY rate, decreasing from 40.69 in 1990 to 37.65 in 2021. In 2021, the female DALY rate is lower than that of males (**Table 2**). At the SDI level, only the High SDI region has experienced an increase in the diabetes DALY rate, while the other four SDI regions have all shown a decline, with the Low SDI region exhibiting the most pronounced decrease (**Table 2**, **Figure 2C**, **Supplementary Figures S3**, **S4**). Subgroup analysis by geographical region indicates that Oceania has the highest DALY rate and displays an increasing trend, rising from 7,025 per 100,000 in 1990 to 80.57 per 100,000 in 2021 (**Table 2**). At the national level, India reports the highest total diabetes DALYs and an increasing trend, rising from 135.04×10³ years in 1990 to 150.73×10³ years in 2021. Conversely, China’s total diabetes DALYs have decreased, falling from the second highest in 1990 (119.81×10³ years) to the third highest in 2021 (59.29×10³ years) (**Figures 3C**, **4C**, **Supplementary Tables S5**, **S6**).

### 3.4 Association between SDI and global incidence and death rates of diabetes in children and adolescents

**Figure 5:**
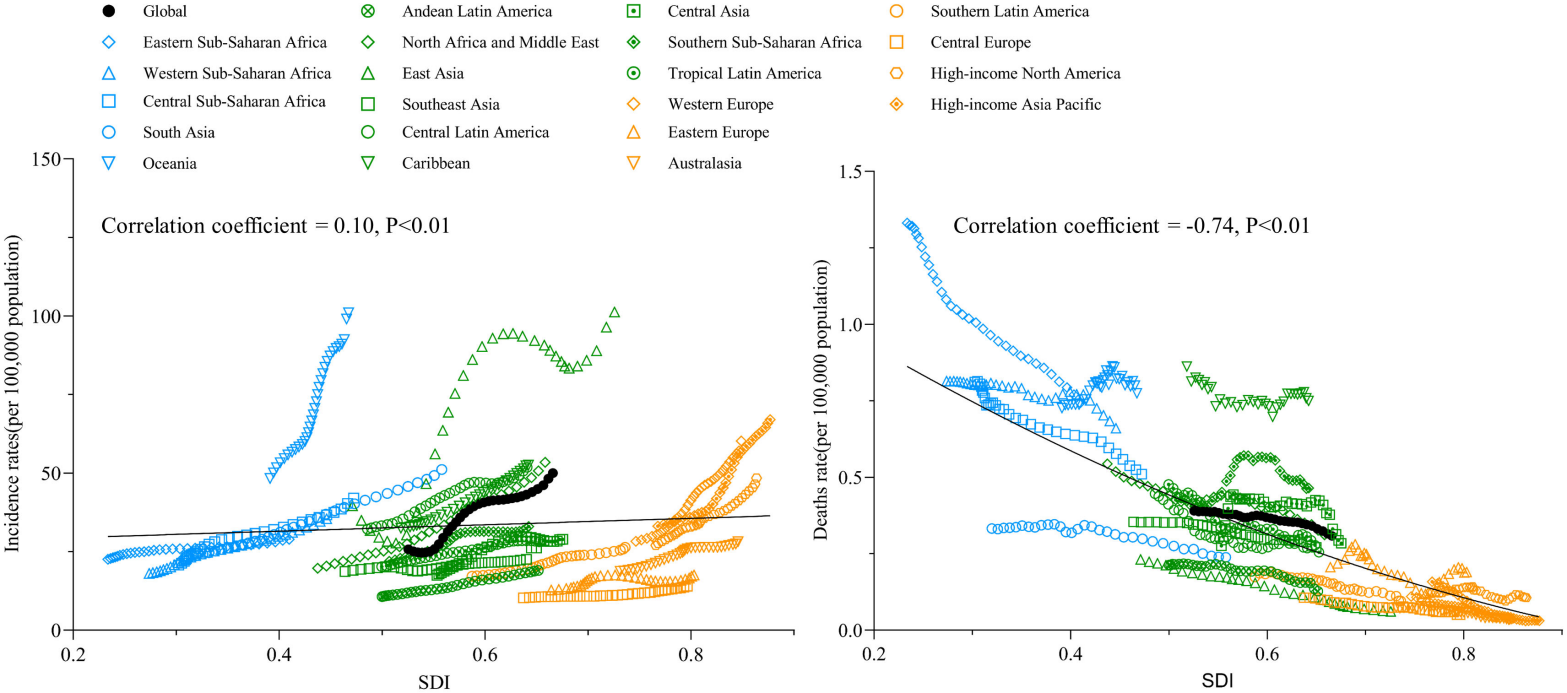
The change trends and correlation analyses of incidence rate and death rate with SDI from 1990 to 2021.

We evaluated the association between trends in the SDI across 21 global regions from 1990 to 2021 and the incidence and death rates of diabetes in children and adolescents. The results reveal that both global and regional diabetes incidence rates are significantly positively associated with SDI (correlation coefficient = 0.10, P < 0.01), whereas diabetes-related death rates are significantly negatively associated with SDI (correlation coefficient = -0.74, P < 0.01) (**Figure 5**).

### 3.5 Age distribution of diabetes incidence among children and adolescents

**Figure 6:**
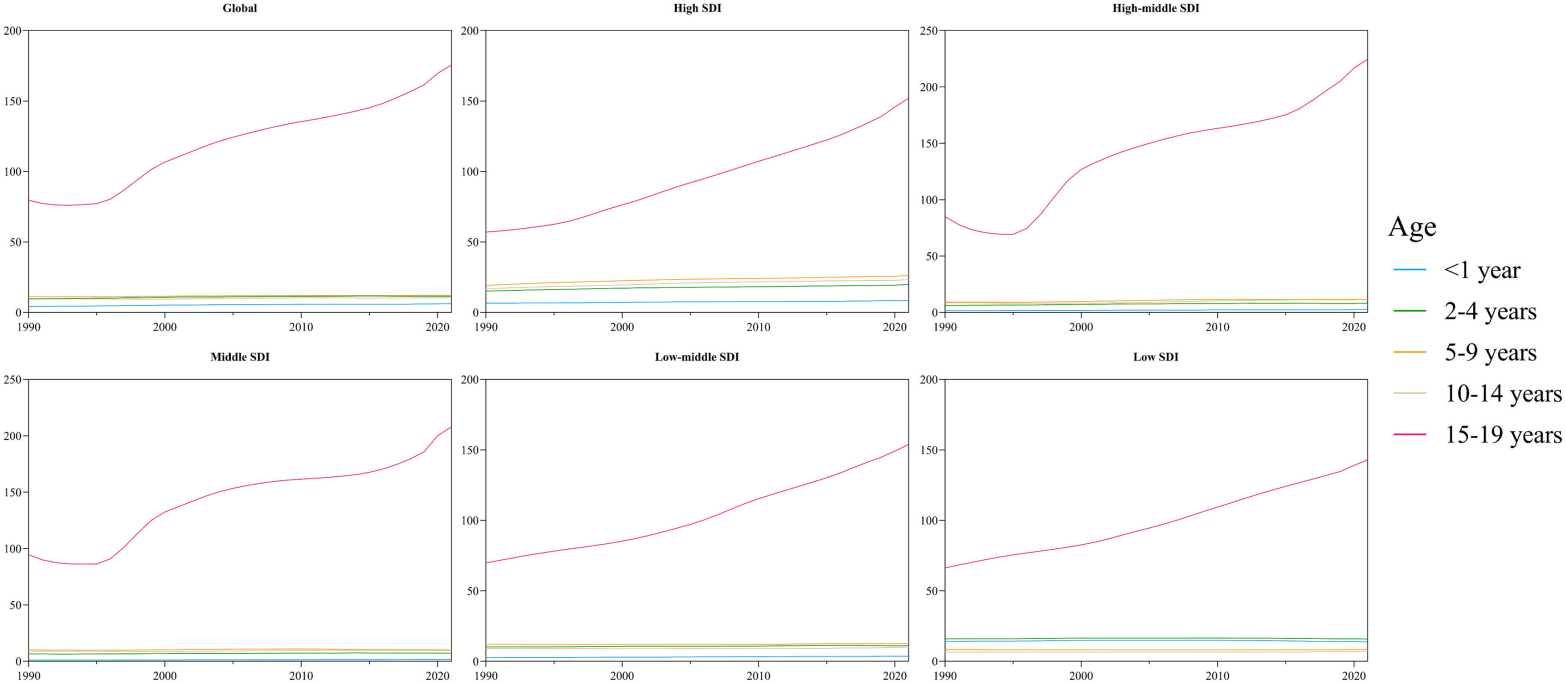
The incidence of cases of diabetes in different age groups from 1990 to 2021.

We examined the incidence rates of diabetes across five distinct age groups (< 1 year, 2 to 4 years, 5 to 9 years, 10 to 14 years, and 15 to 19 years) both globally and within various regions from 1990 to 2021. The results reveal that both globally and across the five SDI regions, the highest incidence rate is observed in the 15 – 19 year age group, which demonstrates a significant upward trend in diabetes incidence (**Figure 6**, **Supplementary Material 3**). In addition, the mortality and DALY rates for type 1 diabetes patients under 1 year of age are significantly higher than those in other age groups, despite this group having the lowest incidence rate. The highest incidence is observed among adolescents aged 10 – 14 years (**Supplementary Figure S6**). Notably, due to the research framework adopted by the GBD, the incidence, mortality, and DALY rates for type 2 diabetes in patients under 15 years of age are all reported as zero (**Supplementary Material 4**).

### 3.6 Risk factors contributing to diabetes-related death in children and adolescents worldwide

**Figure 7:**
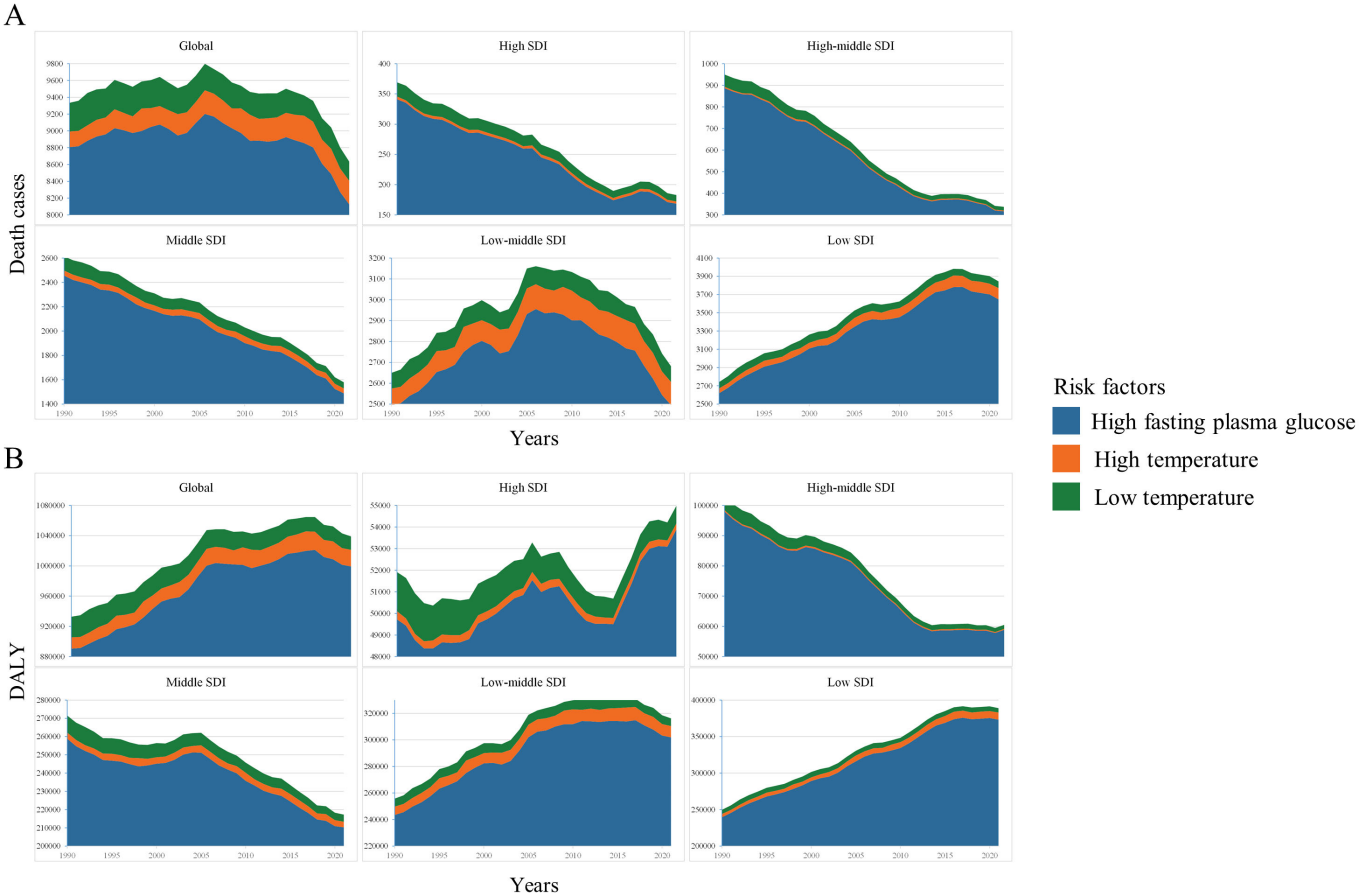
Risk factors contributing to diabetes-related death **(A)** and DALY **(B)**.

We identified three primary risk factors contributing to diabetes-related death and DALYs in children and adolescents: high fasting blood glucose, high temperatures, and low temperatures. Among these risk factors, elevated fasting blood glucose is the predominant contributor to diabetes-related death and DALYs in children and adolescents, both globally and across various regions from 1990 to 2021. In High SDI, High-middle SDI, and Middle SDI regions, low temperatures rank as the second most significant risk factor. In Low-middle SDI and Low SDI regions, high temperatures are identified as the second most significant risk factor (**Figures 7A, B**).

### 3.7 Projecting trends in global childhood and adolescent diabetes incidence and mortality over the next 15 years with ARIMA models

As shown in **Supplementary Figure S7** and **Supplementary Figure S8**, it is projected that the current trends in the incidence and mortality rates of diabetes in children and adolescents worldwide will persist over the next 15 years, with incidence increasing and mortality decreasing.

## 4 Discussion

This study presents the incidence, mortality, and DALY data for diabetes in children and adolescents, both globally and regionally, from 1990 to 2021, using the GBD 2021 database. Additionally, it examines the risk factors associated with diabetes-related mortality within this demographic. The findings indicate that between 1990 and 2021, there have been substantial changes in the burden and epidemiological trends of diabetes among children and adolescents, both globally and regionally. Notably, the global burden of diabetes within this age group has continued to rise.

Between 1990 and 2021, the incidence rate of diabetes among children and adolescents has exhibited a general increase, with a notable rise of 94.0% in global incidence over this period. Recent research attributes these trends to factors such as obesity, familial predisposition, and sedentary lifestyles (21). This increase may reflect broader trends including global population growth, demographic aging, dietary changes, and shifts in lifestyle over the past 32 years (22). Regions with higher Socio-Demographic Index (SDI) scores have demonstrated higher incidence rates compared to regions with lower SDI scores, showing a significant positive correlation between SDI and incidence rates. This pattern aligns with previous perspectives that classify diabetes as a “disease of affluence,” characterized by distinctive modern attributes. Residents of high SDI regions frequently encounter overnutrition and reduced physical activity, contributing to elevated obesity rates among children and adolescents, which is a well-established risk factor for diabetes (23–25). Additionally, this trend may be partly attributable to more advanced diabetes diagnosis and registration systems in high SDI regions (14).

It is encouraging to note that over the past 31 years, the mortality rate and DALYs associated with diabetes in children and adolescents have declined globally. In regions with high SDI scores, mortality rates, and DALYs are markedly lower than in regions with low SDI scores. Correlation analysis further reveals a significant negative correlation between SDI and mortality rates. This trend can be attributed to advancements in basic healthcare coverage globally and the superior medical services available in high SDI regions, which enable more effective and timely diagnosis and treatment of diabetes in children and adolescents (26).

Diabetes is acknowledged as a complex, multifactorial condition influenced by genetic, metabolic, and environmental factors. The precise pathogenesis of diabetes remains elusive, and the exact roles of these factors in the onset and progression of the disease in children and adolescents warrant further investigation (27). Elevated fasting blood glucose is unequivocally the principal risk factor for diabetes-related mortality among children and adolescents. Numerous studies have demonstrated that elevated blood glucose levels in diabetic patients are associated with an increased risk of complications, including coronary heart disease and stroke (28, 29). Diabetic ketoacidosis and hyperosmolar hyperglycemic syndrome, both mediated by elevated blood glucose levels, are also major contributors to diabetes-related mortality (30, 31). Additionally, our analysis revealed that both high and low temperatures are risk factors for diabetes-related mortality in children and adolescents worldwide, with varying impacts depending on SDI regions. One study indicates that rising temperatures may lead to an accumulation of reactive substances due to chronic inflammation, thereby contributing to diabetes complications, with NADPH oxidase potentially playing a significant role (32). Furthermore, an observational study conducted in inland and coastal regions of China identified a significant correlation between environmental temperatures and diabetes mortality (33).

This study offers several significant advantages. Firstly, by leveraging the high-quality evidence and methodological framework provided by GBD 2021, we have accurately reported the incidence and mortality rates of diabetes among children and adolescents, both globally and regionally, from 1990 to 2021. Secondly, in comparison to existing research, our study provides an innovative analysis of the epidemiological status of diabetes in children and adolescents (under 20 years), grounded in current trends and utilizing the most recent data from GBD 2021, thus ensuring the timeliness of our conclusions.

Nevertheless, this study has several limitations. Firstly, the results are based on aggregated data from the GBD study, and the accuracy of this data is contingent upon the quality of data reported by different countries. Certain countries and regions may have a substantial number of undiagnosed cases of diabetes among children and adolescents, coupled with insufficient data on related risk factors, which can undermine the accuracy of the findings. Secondly, The aggregate-level data provided by the GBD prevent the use of conventional epidemiological methods, such as survival analysis or regression analysis, to explore potential associations between individual indicators and diseases, which may introduce bias. Thirdly, the study is limited by the absence of some critical data, which precluded the use of age standardization and thereby constrained our ability to comprehensively describe global epidemiological trends of diabetes in children and adolescents. This limitation may have diminished the overall completeness of the results.

## 5 Conclusions

Over the 32-year period from 1990 to 2021, the global burden of diabetes among children and adolescents remained substantial. Specifically, the incidence rates of both type 1 and type 2 diabetes exhibited an overall upward trend. Although global mortality and DALYs associated with diabetes have declined, this decrease has been primarily driven by improvements in type 1 diabetes outcomes. In contrast, both the mortality rate and DALYs related to type 2 diabetes have actually increased. This suggests that diabetes will continue to pose a serious threat to the health of children and adolescents worldwide. Therefore, healthcare institutions must develop more cost-effective and targeted strategies to alleviate the adverse impacts of diabetes on young populations and to reduce the associated socioeconomic burden.

## Data Availability

The original contributions presented in the study are included in the article/**Supplementary Material**. Further inquiries can be directed to the corresponding author.

## Supplementary material

Supplementary Material 1 Top 20 countries or areas with the highest number of incidence, mortality and DALYs, 1990 or 2021.

Supplementary Material 2 The global burden situation based on the grouping criterion of diabetes types and the ARIMA analysis results.

Supplementary Material 3 The global burden of disease based on age groups.

Supplementary Material 4 The global burden of disease based on age groups and diabetes groups.
