# Supplementary material for: Analysis of the global burden of diabetes and attributable risk factor in children and adolescents across 204 countries and regions from 1990 to 2021
Source: Front Endocrinol (Lausanne). 2025 Sep 8;16:1587055. doi: 10.3389/fendo.2025.1587055 (PMC12450666; doi:10.3389/fendo.2025.1587055)
Supplement: Supplementary Material 4 — The global burden of disease based on age groups and diabetes groups. [file DataSheet4.docx]

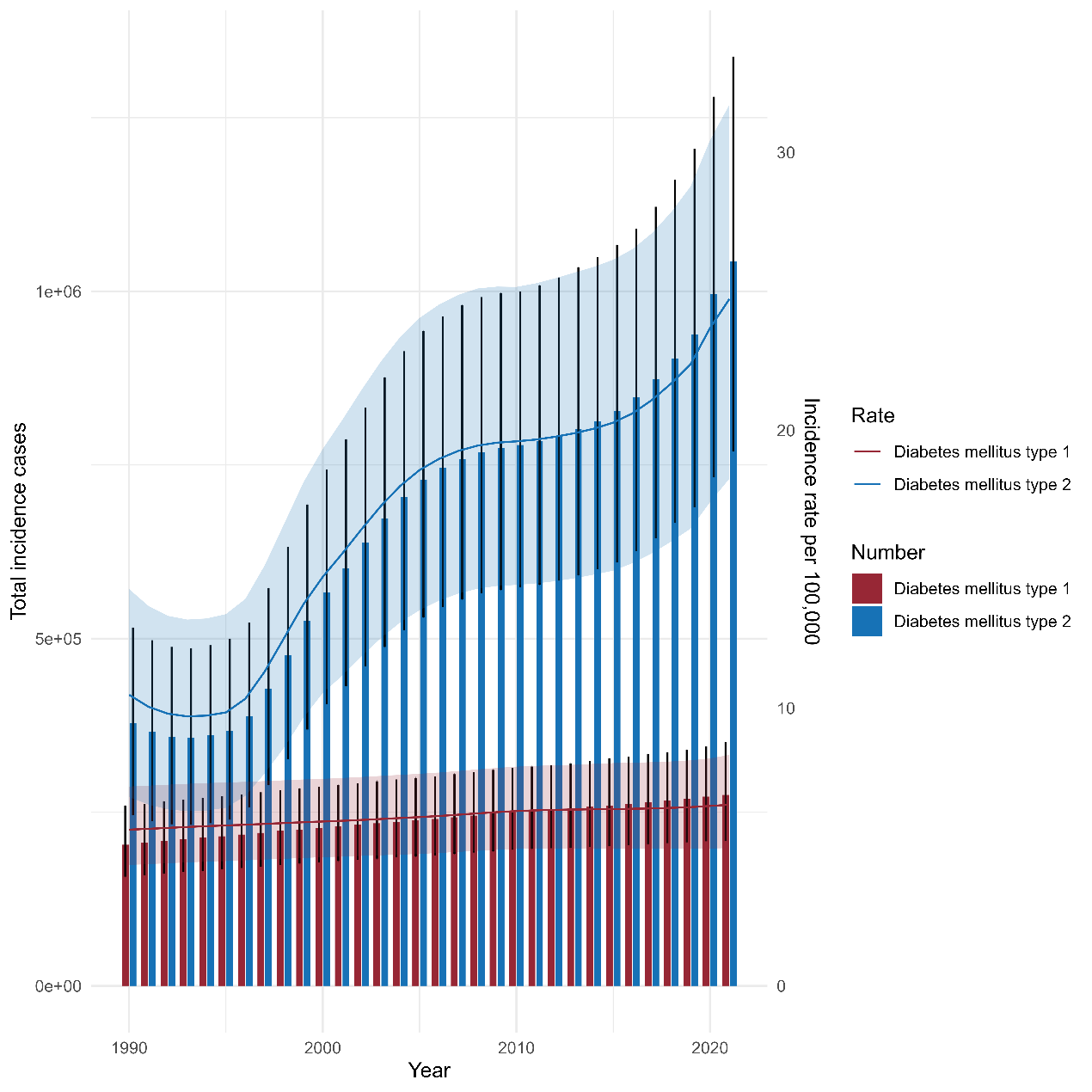


Figure S1- The change trends of diabetes’s incidence cases and incidence rate per 100,000 from 1990 to 2021.
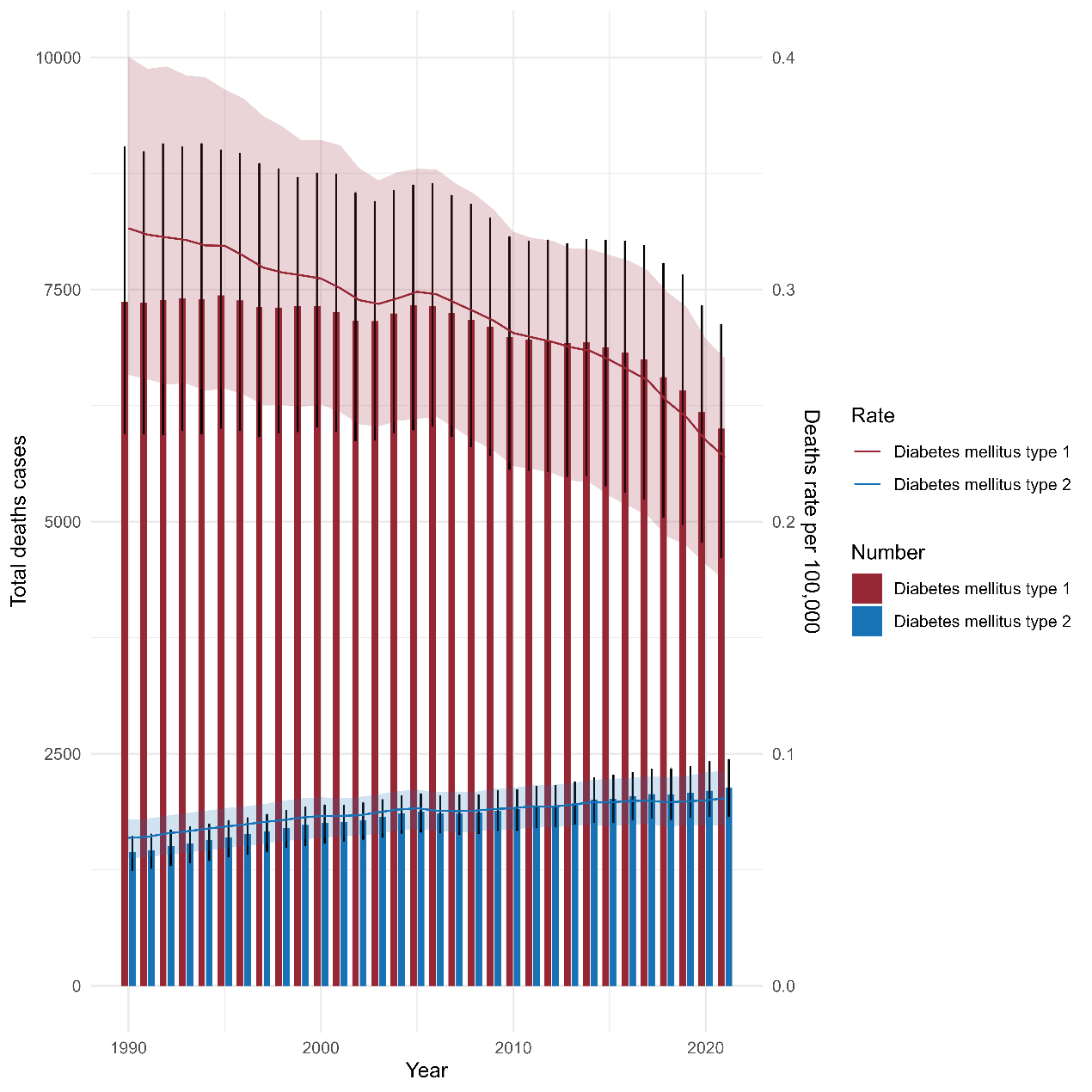


Figure S2- The change trends of diabetes’s deaths cases and deaths rate per 100,000 from 1990 to 2021.


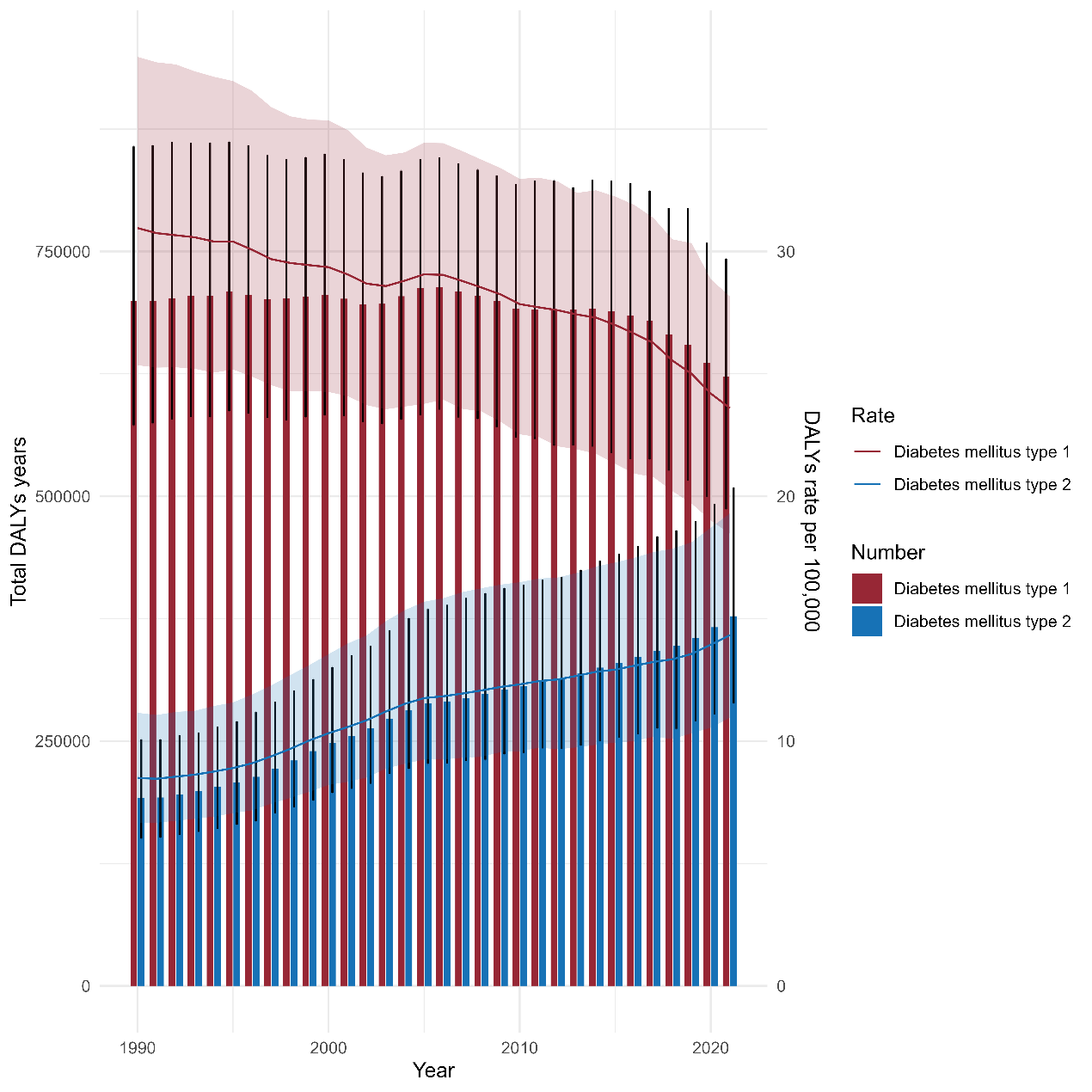


Figure S3- The change trends of diabetes’s DALYs years and DALYs rate per 100,000 from 1990 to 2021.


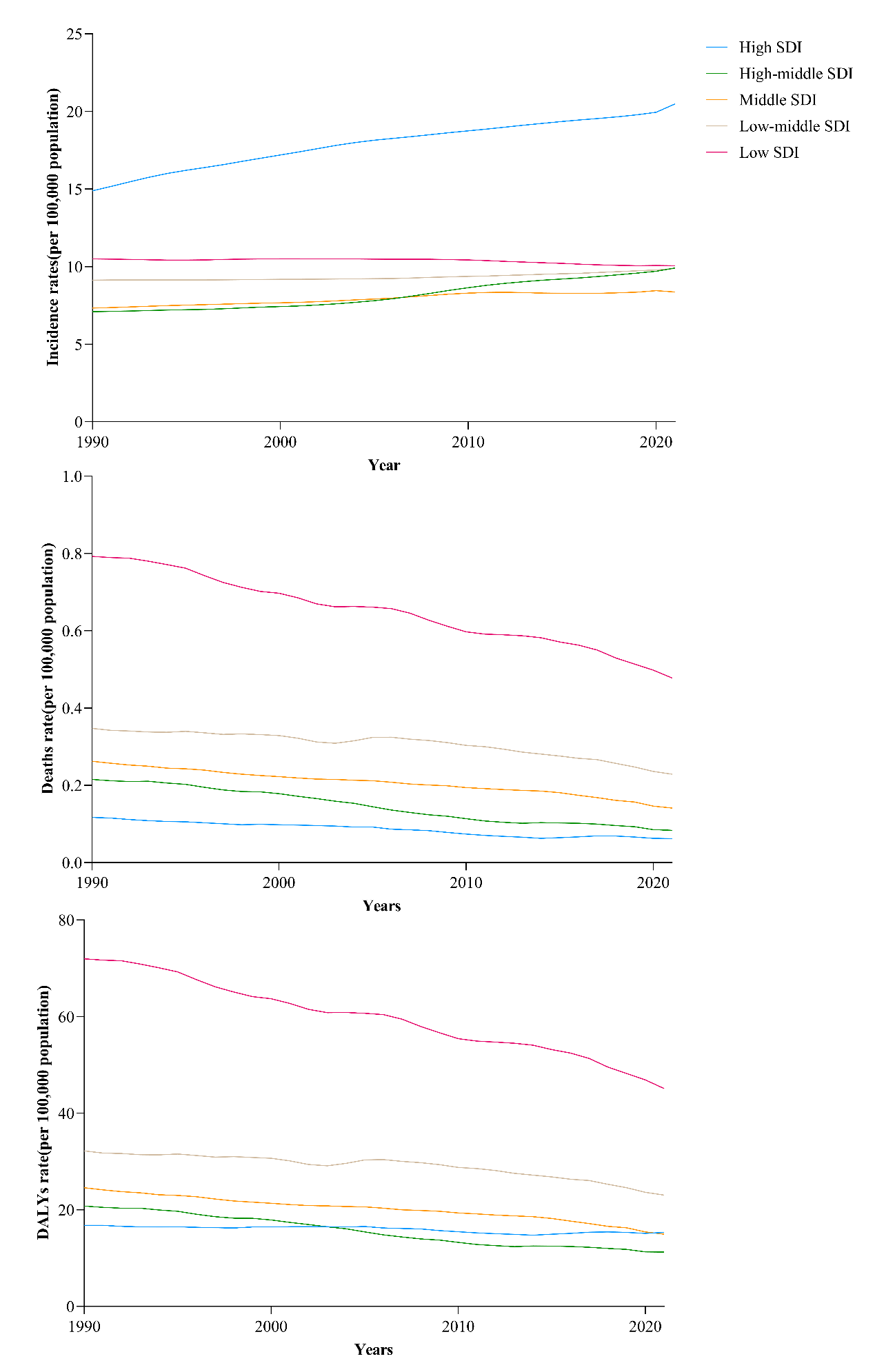


Figure S4- Trends from 1990 to 2021 in the death rate of type 1 diabetes in five SDI regions.


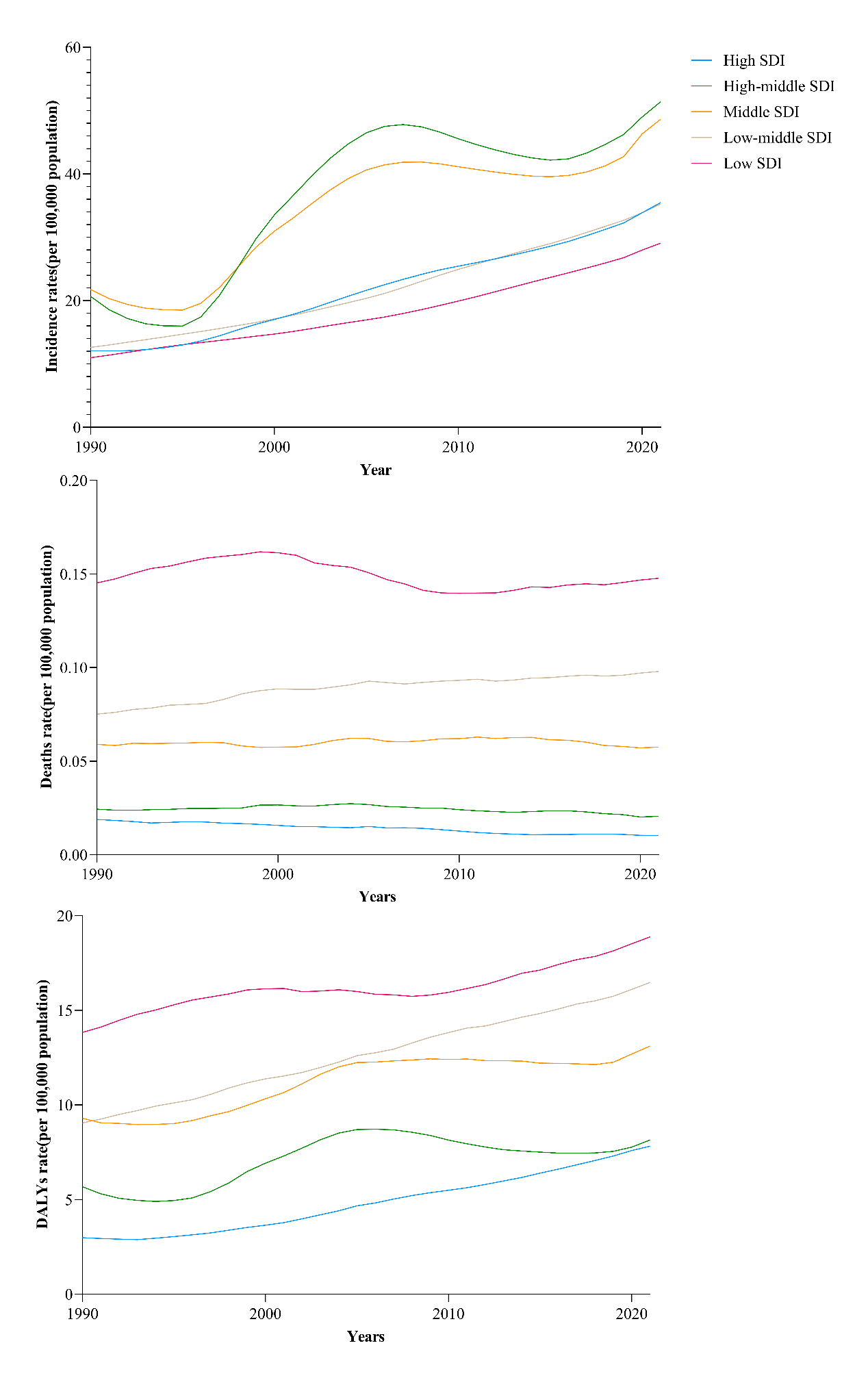


Figure S5- Trends from 1990 to 2021 in the death rate of type 2 diabetes in five SDI regions.


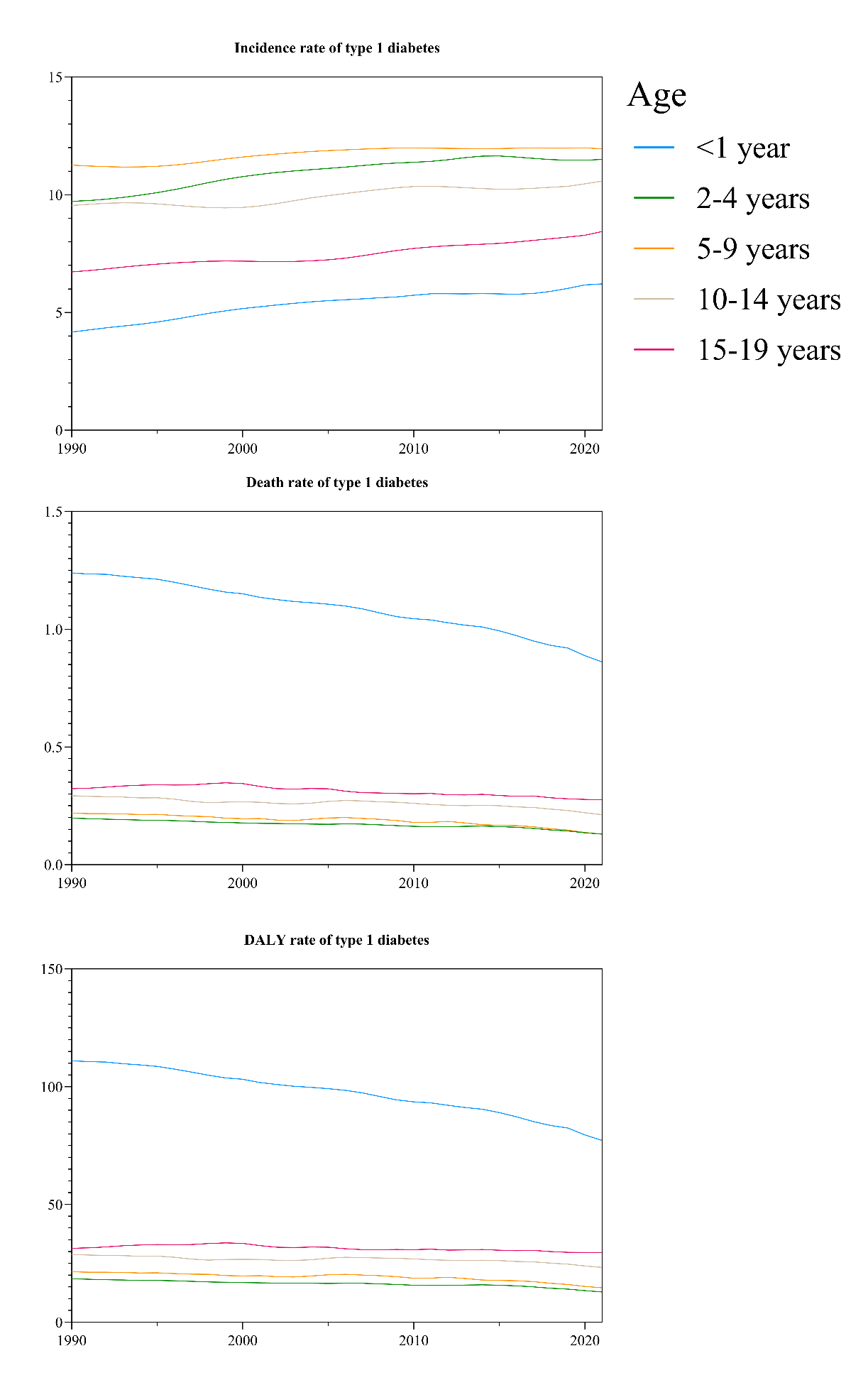


Figure S6- The incidence rate, death rate and DALY rate of type 1 diabetes in different age groups from 1990 to 2021.


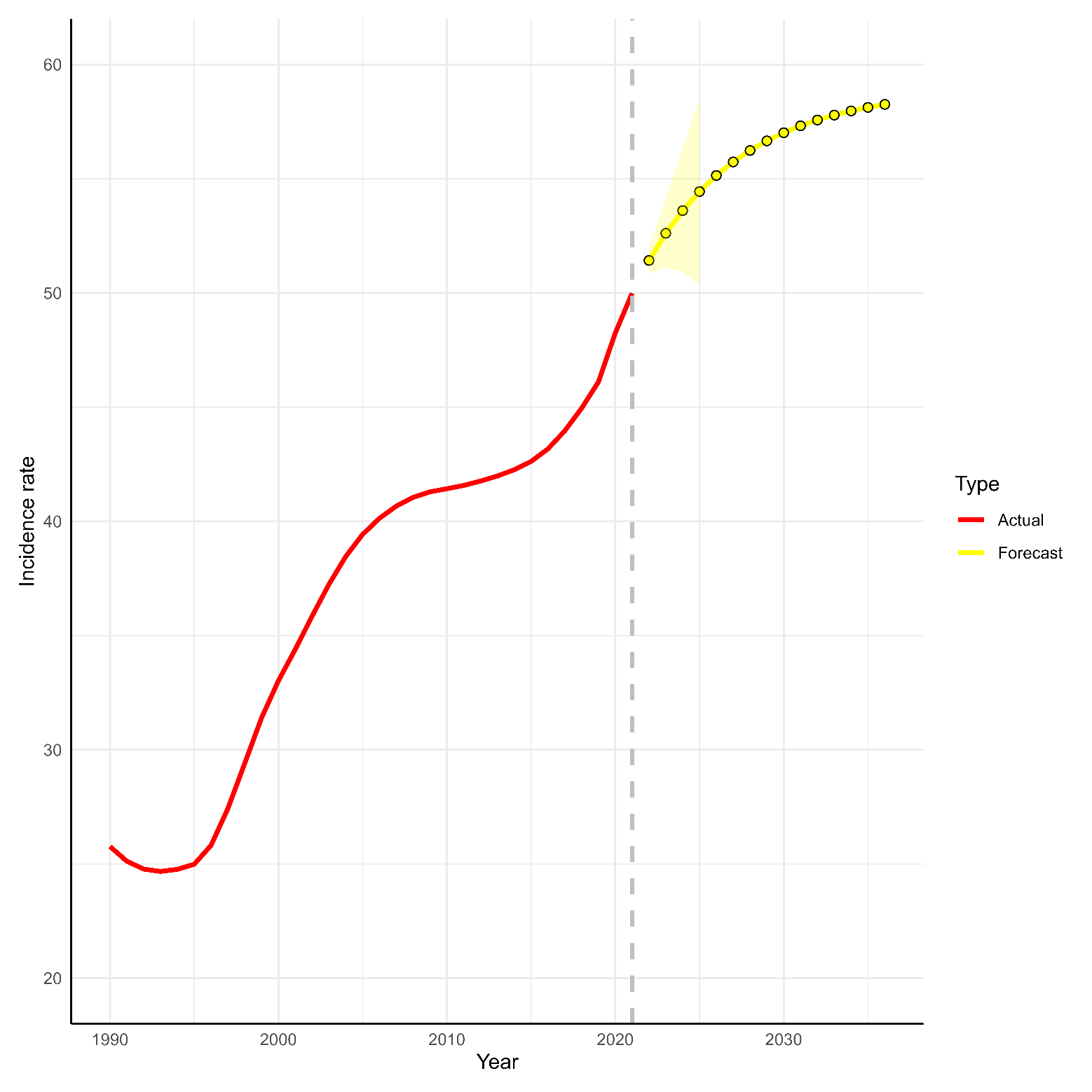


Figure S7- Global prediction of diabetes incidence rate among children and adolescents in the next 15 years.


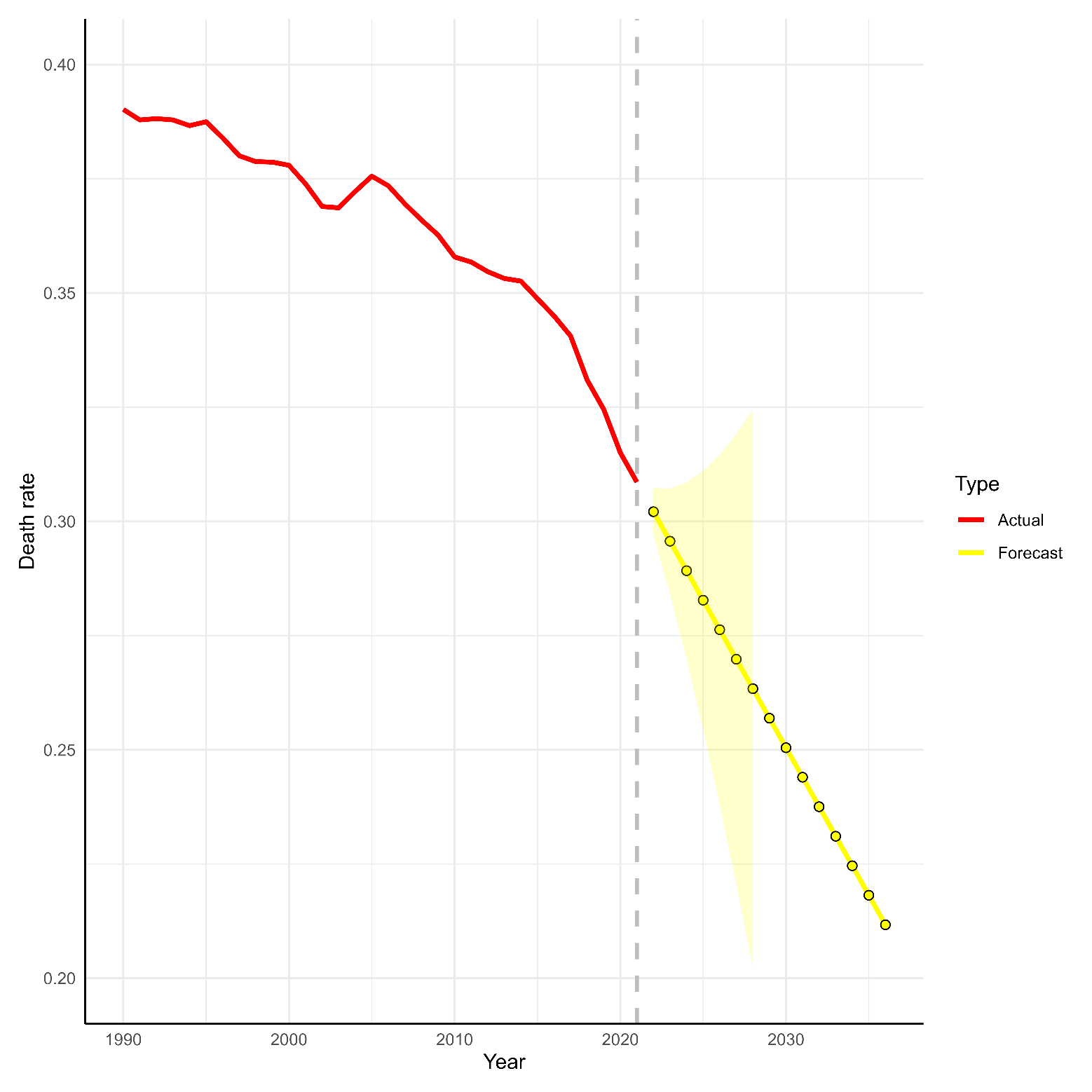


Figure S8- Global prediction of diabetes death rate among children and adolescents in the next 15 years.
