# Supplementary material for: Analysis of the global burden of diabetes and attributable risk factor in children and adolescents across 204 countries and regions from 1990 to 2021
Source: Front Endocrinol (Lausanne). 2025 Sep 8;16:1587055. doi: 10.3389/fendo.2025.1587055 (PMC12450666; doi:10.3389/fendo.2025.1587055)
Supplement: Supplementary file 5 [file Table1.docx]

Table S1. Top 20 countries or territories with most incidence cases in 1990

| **Location** | **Incidence case** |
| --- | --- |
| People's Republic of China | 177561.2644 |
| Republic of India | 93205.34521 |
| United States of America | 19374.31386 |
| United Mexican States | 18204.97385 |
| People's Republic of Bangladesh | 18194.65626 |
| Republic of Indonesia | 15912.9777 |
| Islamic Republic of Pakistan | 14763.69065 |
| Federative Republic of Brazil | 13681.60113 |
| Japan | 10040.89166 |
| Federal Democratic Republic of Ethiopia | 8122.185364 |
| Federal Republic of Nigeria | 7931.225583 |
| Republic of the Philippines | 7640.137939 |
| Republic of Korea | 6250.964554 |
| Russian Federation | 5655.367524 |
| Republic of the Union of Myanmar | 4817.836451 |
| Islamic Republic of Iran | 4765.409942 |
| Republic of Turkey | 4704.993937 |
| Republic of Italy | 4693.03557 |
| Republic of South Africa | 4321.680734 |
| Socialist Republic of Viet Nam | 4318.659122 |

Table S2. Top 20 countries or territories with most incidence cases in 2021

| **Location** | **Incidence case** |
| --- | --- |
| People's Republic of China | 342120.9 |
| Republic of India | 243000.6 |
| Islamic Republic of Pakistan | 53726.33 |
| People's Republic of Bangladesh | 43792.14 |
| United States of America | 38316.6 |
| Federal Republic of Nigeria | 33168.96 |
| United Mexican States | 25425.91 |
| Republic of Indonesia | 22053.56 |
| Republic of Iraq | 18891.62 |
| Federal Democratic Republic of Ethiopia | 18596.72 |
| Democratic Republic of the Congo | 18213.7 |
| Islamic Republic of Afghanistan | 18094.74 |
| Federative Republic of Brazil | 18060.39 |
| Arab Republic of Egypt | 16529.94 |
| United Kingdom of Great Britain and Northern Ireland | 15756.05 |
| Japan | 10905.15 |
| Republic of the Philippines | 10554.77 |
| Kingdom of Morocco | 10443.16 |
| Republic of Mali | 10182.84 |
| Republic of Sudan | 9190.794 |

Table S3. Top 20 countries or territories with most death cases in 1990

| **Location** | **Death case** |
| --- | --- |
| Republic of India | 1316.632259 |
| People's Republic of China | 1023.137727 |
| Federal Democratic Republic of Ethiopia | 519.4431621 |
| Federal Republic of Nigeria | 370.537943 |
| Federative Republic of Brazil | 320.4183081 |
| Republic of Indonesia | 309.4007467 |
| People's Republic of Bangladesh | 273.4918019 |
| United Mexican States | 245.5076216 |
| United Republic of Tanzania | 217.5443333 |
| Republic of Turkey | 196.537336 |
| Islamic Republic of Pakistan | 184.1374808 |
| Arab Republic of Egypt | 169.1805889 |
| Democratic Republic of the Congo | 166.4664806 |
| Republic of the Union of Myanmar | 132.3241848 |
| Republic of the Philippines | 129.1656154 |
| Republic of Mozambique | 121.0836064 |
| Russian Federation | 96.60257868 |
| Republic of Malawi | 92.55272193 |
| United States of America | 92.28562353 |
| Islamic Republic of Iran | 91.78914101 |

Table S4. Top 20 countries or territories with most death cases in 2021

| **Location** | **Death case** |
| --- | --- |
| Republic of India | 964.6402754 |
| Federal Republic of Nigeria | 802.5258007 |
| Islamic Republic of Pakistan | 421.2244373 |
| Federal Democratic Republic of Ethiopia | 383.4170596 |
| United Republic of Tanzania | 262.6430525 |
| Republic of Indonesia | 249.0739883 |
| Democratic Republic of the Congo | 240.3090492 |
| People's Republic of Bangladesh | 218.3347126 |
| People's Republic of China | 192.2235182 |
| Republic of Mozambique | 178.9830002 |
| Republic of Uganda | 166.5935675 |
| Arab Republic of Egypt | 165.5220382 |
| Federative Republic of Brazil | 157.9827963 |
| Republic of the Philippines | 153.271726 |
| Islamic Republic of Afghanistan | 141.819818 |
| United Mexican States | 133.6672221 |
| Federal Republic of Somalia | 133.0986602 |
| Republic of Cameroon | 117.5484531 |
| Republic of Madagascar | 106.2967908 |
| Republic of Mali | 100.5736465 |

Table S5. Top 20 countries or territories with most DALY in 1990

| **Location** | **DALY** |
| --- | --- |
| Republic of India | 135035.6901 |
| People's Republic of China | 119808.0187 |
| Federal Democratic Republic of Ethiopia | 46800.29874 |
| Federal Republic of Nigeria | 33137.38576 |
| Federative Republic of Brazil | 30690.51069 |
| Republic of Indonesia | 29026.88534 |
| People's Republic of Bangladesh | 27919.9531 |
| United Mexican States | 26002.72125 |
| United Republic of Tanzania | 19915.61319 |
| Islamic Republic of Pakistan | 19312.37688 |
| Republic of Turkey | 18173.93326 |
| Arab Republic of Egypt | 15193.14885 |
| Democratic Republic of the Congo | 15102.5867 |
| United States of America | 15032.30744 |
| Republic of the Philippines | 13824.95487 |
| Republic of the Union of Myanmar | 11466.58658 |
| Republic of Mozambique | 11042.64388 |
| Russian Federation | 9527.431661 |
| Islamic Republic of Iran | 9287.2036 |
| Republic of Uganda | 8408.566525 |

Table S6. Top 20 countries or territories with most DALY in 2021

| **Location** | **DALY** |
| --- | --- |
| Republic of India | 150726.4 |
| Federal Republic of Nigeria | 76665.06 |
| People's Republic of China | 59293.69 |
| Islamic Republic of Pakistan | 48680.24 |
| Federal Democratic Republic of Ethiopia | 39255.74 |
| People's Republic of Bangladesh | 30860.81 |
| Republic of Indonesia | 25688.24 |
| United Republic of Tanzania | 25671.65 |
| Democratic Republic of the Congo | 25104.26 |
| United States of America | 23905.42 |
| Federative Republic of Brazil | 18316.93 |
| United Mexican States | 18096.38 |
| Arab Republic of Egypt | 17366.82 |
| Republic of Mozambique | 17065.59 |
| Republic of Uganda | 16530.05 |
| Republic of the Philippines | 15437.87 |
| Islamic Republic of Afghanistan | 15093.87 |
| Federal Republic of Somalia | 12645.36 |
| Republic of Cameroon | 11733.19 |
| Republic of Mali | 11282.57 |
